# Supplementary material for: Chemical bonding concepts emerge naturally from maximally entangled atomic orbitals
Source: Nat Commun. 2026 May 27;17:4732. doi: 10.1038/s41467-026-73527-w (PMC13216281; doi:10.1038/s41467-026-73527-w)
Supplement: Supplementary file 1 — Supplementary Information [file 41467_2026_73527_MOESM1_ESM.pdf]

Supplementary Information for:
"Chemical bonding concepts emerge naturally from maximally entangled atomic orbitals"

Lexin Ding,[1,2] Eduard Matito,[3] and Christian Schilling[1,2,*]

[1] *Faculty of Physics, Arnold Sommerfeld Centre for Theoretical Physics (ASC), Ludwig-Maximilians-Universität München, Theresienstr. 37, 80333 München, Germany*
[2] *Munich Center for Quantum Science and Technology (MCQST), Schellingstrasse 4, 80799 München, Germany*
[3] *Donostia International Physics Center (DIPC), Donostia 20018 Euskadi, Spain*

In this document, we provide further details that complement the results presented in the main text. First, we discuss the derivations and properties of the cost function used to construct the maximally entangled atomic orbitals (MEAOs). Second, we present additional graphics showing the consistent recovery of orbital hybridization and Lewis structures in the MEAO framework. Third, an extended analysis on the LiH dissociation is presented. Finally, we recap the definitions of the bonding indices we used for comparison.

Supplementary Note 1: Properties and derivatives of the objective function F_{MEAO}

The relevant spin sector of the two-particle reduced density matrix (2RDM) Γ of a wavefunction $|\Psi\rangle$ in an orbital basis $\mathcal{B}$ is defined as

$$\Gamma(\mathcal{B})^{i\bar{j}}_{k\bar{l}} = \langle \Psi | f^\dagger_{i\uparrow} f^\dagger_{j\downarrow} f_{l\downarrow} f_{k\uparrow} | \Psi \rangle, \tag{1}$$

where $f^{(\dagger)}_{i\sigma}$ is the fermionic annihilation (creation) operator that annihilates (creates) a spin-σ electron in the i-th spatial orbital. Here, and in the following, we interpret orbital rotations as active transformations which also motivates our notation $\Gamma \equiv \Gamma(\mathcal{B})$. The objective function for maximizing the entanglement between connected orbitals then takes the form

$$F_{\mathrm{MEAO}}(\mathcal{B}) = \sum_{i<j} |\Gamma(\mathcal{B})^{i\bar{i}}_{j\bar{j}}|^2 + |\Gamma(\mathcal{B})^{i\bar{j}}_{j\bar{i}}|^2, \tag{2}$$

where the sum in (2) excludes pairs (i,j) of indices belonging to the same atomic center, and i $(\bar{i})$ denotes the i-th spin-up (-down) orbital.

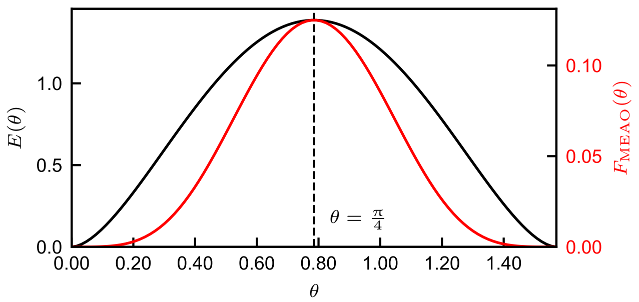

Suppl. Fig. 1. **Entanglement and the cost function F_{MEAO} for constructing the maximally entangled atomic orbitals evaluated for the rotated state, Eq.** (5). Entanglement between ψ_{L} and ψ_{R} (left axis, black) and the cost function F_{MEAO} (right axis, red) are shown as functions of the orbital rotational parameter θ.

* c.schilling@physik.uni-muenchen.de

For the quantum state of an idealized single two-center covalent bond,

$$|\Psi_{\text{bond}}\rangle = |\uparrow\downarrow\rangle_{\phi} \otimes |0\rangle_{\bar{\phi}}, \tag{3}$$

where ϕ and $\bar{\phi}$ are the bonding and antibonding orbitals, respectively, maximizing F_{MEAO} leads to the same set of orbitals that maximize the left-right entanglement. To show this, we consider the following orbital parameterization.

$$\begin{aligned} \psi_{\text{L}} &= \cos(\theta)\phi + \sin(\theta)\overline{\phi}, \\ \psi_{\text{R}} &= -\sin(\theta)\phi + \cos(\theta)\overline{\phi}. \end{aligned} \tag{4}$$

The state $|\Psi_{\text{bond}}\rangle$ can then be re-expressed as

$$\begin{aligned} |\Psi_{\text{bond}}(\theta)\rangle = &\cos^2(\theta)|\uparrow\downarrow\rangle_{\psi_{\text{L}}} \otimes |0\rangle_{\psi_{\text{R}}} + \sin^2(\theta)|0\rangle_{\psi_{\text{L}}} \otimes |\uparrow\downarrow\rangle_{\psi_{\text{R}}} \\ &+ \cos(\theta)\sin(\theta)|\uparrow\rangle_{\psi_{\text{L}}} \otimes |\downarrow\rangle_{\psi_{\text{R}}} - \cos(\theta)\sin(\theta)|\downarrow\rangle_{\psi_{\text{L}}} \otimes |\uparrow\rangle_{\psi_{\text{R}}}. \end{aligned} \tag{5}$$

The entanglement E between the transformed orbitals ψ_{L} and ψ_{R} is given by

$$\begin{aligned} E(\theta) &= S(\hat{\rho}_{L/R}(\theta)) = -2\cos^2\theta\log(\cos^2\theta) - 2\sin^2\theta\log(\sin^2\theta), \\ \hat{\rho}_{\text{L/R}}(\theta) &= \text{Tr}_{\text{R/L}}[|\Psi_{\text{bond}}(\theta)\rangle\langle\Psi_{\text{bond}}(\theta)|], \\ S(\hat{\rho}) &= -\text{Tr}[\hat{\rho}\log(\hat{\rho})]. \end{aligned} \tag{6}$$

Similarly, the cost function $F_{\text{MEAO}}(\theta)$ in the transformed basis depends on θ and follows as

$$F_{\text{MEAO}}(\theta) = 2\cos^4\theta\sin^4\theta. \tag{7}$$

Both $E(\theta)$ and $F_{\text{MEAO}}(\theta)$ are maximized at the same value of $\theta = \frac{\pi}{4}$, as shown in Supplementary Figure 1. This shows that $F_{\text{MEAO}}(\theta)$ is a viable cost function to maximize the exact entanglement in this example.

The derivatives of F_{MEAO} with respect to the orbital rotation parameters are now calculated. A Jacobi rotation on the pair (m, n) by an angle θ transforms $\Gamma(\mathcal{B})$ to a new basis $\mathcal{B}'$ as

$$\Gamma(\mathcal{B}')^{ij}_{kl} = \sum_{abcd} \mathbf{J}^{(mn)}(\theta)_{ia}\mathbf{J}^{(mn)}(\theta)_{jb}\mathbf{J}^{(mn)}(\theta)_{kc}\mathbf{J}^{(mn)}(\theta)_{ld}\Gamma(\mathcal{B})^{ab}_{cd}, \tag{8}$$

where

$$J^{(mn)}(\theta)_{ij} = \begin{cases} 1, & i = j, i, j \neq m, n, \\ 0, & i \neq j, \text{and (i}\neq\text{m, n or j}\neq\text{ m, n)} \\ \cos(\theta), & i = m, j = m, \\ \sin(\theta), & i = m, j = n, \\ -\sin(\theta), & i = n, j = m, \\ \cos(\theta), & i = n, j = n. \end{cases} \tag{9}$$

Using these expressions, we can calculate the first derivatives of F with respect to θ_{kl} evaluated at $\theta_{kl} = 0$

$$\begin{aligned} \left.\frac{\partial F_{\text{MEAO}}}{\partial \theta_{kl}}\right|_{\theta_{kl}=0} &= 2\sum_{(i,j)} (\Gamma^{i\bar{i}}_{j\bar{j}}) \left.\frac{\partial \Gamma^{i\bar{i}}_{j\bar{j}}}{\partial \theta_{kl}}\right|_{\theta_{kl}=0} + (\Gamma^{i\bar{j}}_{j\bar{i}}) \left.\frac{\partial \Gamma^{i\bar{j}}_{j\bar{i}}}{\partial \theta_{kl}}\right|_{\theta_{kl}=0}, \\ \left.\frac{\partial \Gamma^{i\bar{i}}_{j\bar{j}}}{\partial \theta_{kl}}\right|_{\theta_{kl}=0} &= \delta_{ik}(\Gamma^{l\bar{i}}_{j\bar{j}} + \Gamma^{i\bar{l}}_{j\bar{j}}) - \delta_{il}(\Gamma^{k\bar{i}}_{j\bar{j}} + \Gamma^{i\bar{k}}_{j\bar{j}}) + \delta_{jk}(\Gamma^{i\bar{i}}_{l\bar{j}} + \Gamma^{i\bar{i}}_{j\bar{l}}) - \delta_{jl}(\Gamma^{i\bar{i}}_{k\bar{j}} + \Gamma^{i\bar{i}}_{j\bar{k}}), \\ \left.\frac{\partial \Gamma^{i\bar{j}}_{j\bar{i}}}{\partial \theta_{kl}}\right|_{\theta_{kl}=0} &= \delta_{ik}(\Gamma^{l\bar{j}}_{j\bar{i}} + \Gamma^{i\bar{j}}_{j\bar{l}}) - \delta_{il}(\Gamma^{k\bar{j}}_{j\bar{i}} + \Gamma^{i\bar{j}}_{j\bar{k}}) + \delta_{jk}(\Gamma^{i\bar{l}}_{j\bar{i}} + \Gamma^{i\bar{j}}_{l\bar{i}}) - \delta_{jl}(\Gamma^{i\bar{k}}_{j\bar{i}} + \Gamma^{i\bar{j}}_{k\bar{i}}), \end{aligned} \tag{10}$$

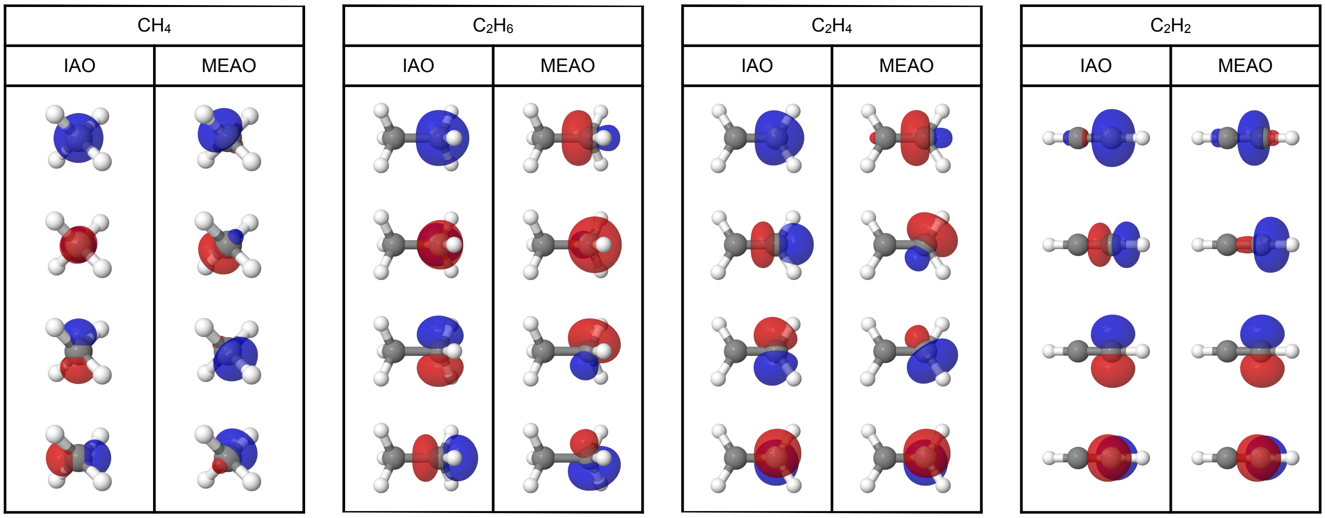

Suppl. Fig. 2. **Orbital isosurfaces of the intrinsic atomic orbitals (IAOs) and the maximally entangled atomic orbitals (MEAOs) in the ground state of CH_4, C_2H_6, C_2H_4, and C_2H_2.** The calculations are performed with the cc-pVDZ basis. Only valence orbitals are shown. Isosurface values are set at 0.2 for CH_4 and 0.1 for C_2H_6, C_2H_4, and C_2H_2. $1s$ orbitals are not presented. Source data are available on Zenodo [1].

and the second derivatives (diagonal elements only) of F with respect to θ_{kl} evaluated at $\theta_{kl} = 0$

$$\begin{aligned}
\left.\frac{\partial^2 F_{\mathrm{MEAO}}}{\partial \theta_{kl}^2}\right|_{\theta_{kl}=0} &= \sum_{(i,j)} 2 \left.\left(\frac{\partial \Gamma^{i\bar{i}}_{j\bar{j}}}{\partial \theta_{kl}}\right)^2\right|_{\theta_{kl}=0} + 2\Gamma^{i\bar{i}}_{j\bar{j}} \left.\frac{\partial^2 \Gamma^{i\bar{i}}_{j\bar{j}}}{\partial \theta_{kl}^2}\right|_{\theta_{kl}=0} \\
&\quad + 2 \left.\left(\frac{\partial \Gamma^{i\bar{j}}_{j\bar{i}}}{\partial \theta_{kl}}\right)^2\right|_{\theta_{kl}=0} + 2\Gamma^{i\bar{j}}_{j\bar{i}} \left.\frac{\partial^2 \Gamma^{i\bar{j}}_{j\bar{i}}}{\partial \theta_{kl}^2}\right|_{\theta_{kl}=0}, \\
\left.\frac{\partial^2 \Gamma^{i\bar{i}}_{j\bar{j}}}{\partial \theta_{kl}^2}\right|_{\theta_{kl}=0} &= -2(\delta_{ik}+\delta_{il}+\delta_{jk}+\delta_{jl})\Gamma^{i\bar{i}}_{j\bar{j}} + 2(\delta_{ik}\Gamma^{l\bar{l}}_{j\bar{j}}+\delta_{il}\Gamma^{k\bar{k}}_{j\bar{j}}+\delta_{jk}\Gamma^{i\bar{i}}_{l\bar{l}}+\delta_{jl}\Gamma^{i\bar{i}}_{k\bar{k}}) \\
&\quad - 2(\delta_{ik}\delta_{jl}+\delta_{jl}\delta_{ik})(\Gamma^{i\bar{j}}_{i\bar{j}}+\Gamma^{i\bar{j}}_{j\bar{i}}+\Gamma^{j\bar{i}}_{i\bar{j}}+\Gamma^{j\bar{i}}_{j\bar{i}}), \\
\left.\frac{\partial^2 \Gamma^{i\bar{j}}_{j\bar{i}}}{\partial \theta_{kl}^2}\right|_{\theta_{kl}=0} &= -2(\delta_{ik}+\delta_{il}+\delta_{jk}+\delta_{jl})\Gamma^{i\bar{j}}_{j\bar{i}} + 2(\delta_{ik}\Gamma^{l\bar{j}}_{j\bar{l}}+\delta_{il}\Gamma^{k\bar{j}}_{j\bar{k}}+\delta_{jk}\Gamma^{i\bar{l}}_{l\bar{i}}+\delta_{jl}\Gamma^{i\bar{k}}_{k\bar{i}}) \\
&\quad - 2(\delta_{ik}\delta_{jl}+\delta_{jl}\delta_{ik})(\Gamma^{i\bar{i}}_{j\bar{j}}+\Gamma^{j\bar{j}}_{i\bar{i}}+\Gamma^{i\bar{j}}_{i\bar{j}}+\Gamma^{j\bar{i}}_{j\bar{i}}).
\end{aligned} \tag{11}$$

When the 2RDM is derived from a mean field wavefunction, the relevant part can be expressed with the one-particle reduced density matrix (1RDM) γ as

$$\Gamma^{i\bar{j}}_{k\bar{l}} = \langle f^\dagger_{i\uparrow} f^\dagger_{j\downarrow} f_{l\downarrow} f_{k\uparrow} \rangle = \gamma^i_k \gamma^{\bar{j}}_{\bar{l}}, \tag{12}$$

which can be substituted into the gradient and second derivative expressions.

Supplementary Note 2: Orbital hybridization in MEAOs

In the main text, we briefly discussed that the MEAO basis automatically recovers the correct hybridization for describing bonding, using the example of C_2H_4, which displays an sp^2 hybridization. This feature of MEAOs holds for various types of hybridization. In Supplementary Figure 2, we present the isosurfaces of IAOs and MEAOs for prototypical molecules with single, double, and triple bonds. The bonding in CH_4 and C_2H_6 is described by an sp^3

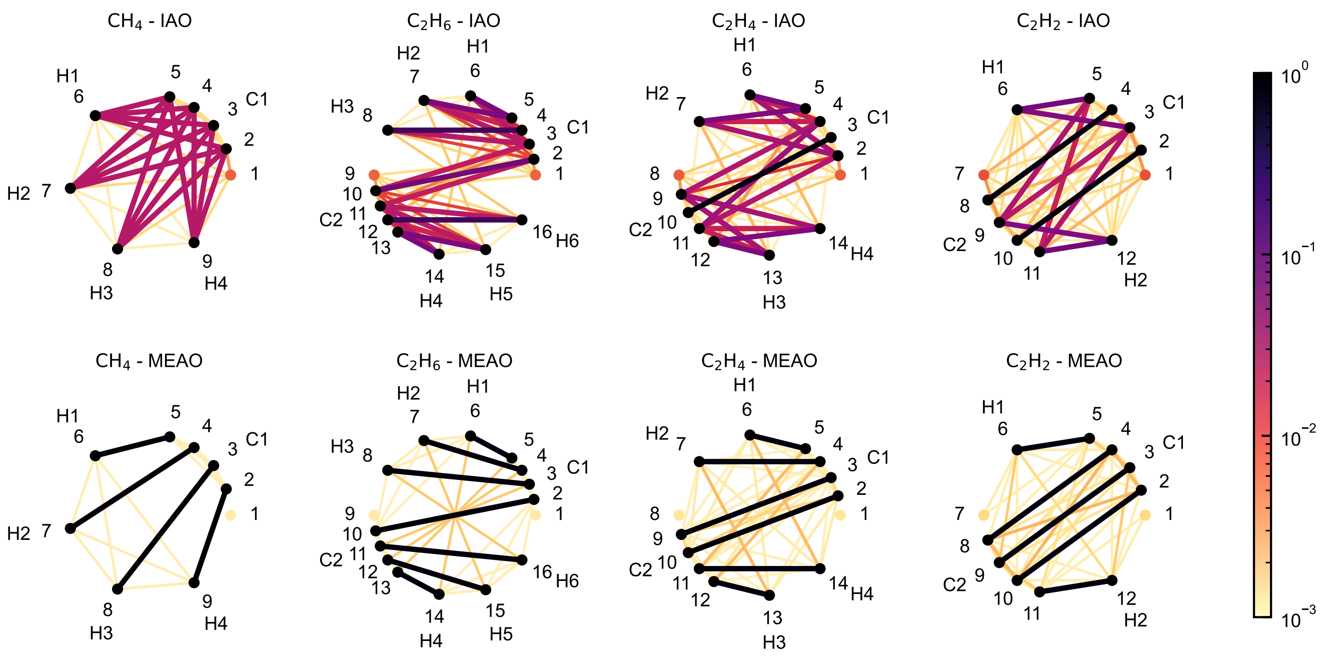

Suppl. Fig. 3. **Orbital correlation graphs of the intrinsic atomic orbitals (IAOs) and the maximally entangled atomic orbitals (MEAOs) in the ground state of CH_4, C_2H_6, C_2H_4, and C_2H_2.** Values of the normalized single orbital entropy $S(\hat{\rho}_i)/\log(4)$ and the normalized orbital-orbital correlation $I_{ij}/\log(16)$ are represented in log-scale by the color of the nodes and edges of the graph, respectively. The calculations are performed with the cc-pVDZ basis. Source data are provided as a Source Data file.

hybridization, where the carbon $2s$ orbital mixes with three $2p$ orbitals to form four hybridized orbitals pointing toward the bonding C and H atoms. The effect of hybridization is clearly observed when comparing the isosurfaces of the IAOs and MEAOs of CH_4 and C_2H_6. The carbon IAOs retain mostly free atomic character, with a clear separation of s and p symmetry, whereas the carbon MEAOs align along the bond paths and point toward the bonding C and H atoms. Similarly, for C_2H_2, the correct sp hybridization is recovered by the MEAOs, where the carbon $2s$ orbital mixes with one carbon $2p$ orbital along the principal axis, while the other two $2p$ orbitals perpendicular to the principal axis remain unchanged after the orbital transformation from IAOs to MEAOs.

In Supplementary Figure 3, we present the corresponding orbital correlation diagrams of the IAOs and MEAOs for the aforementioned molecules. The observations made in the main text for C_2H_4 also apply to other bonding molecules. From IAOs to MEAOs, the correlation between atomic centers is reorganized into maximally correlated orbital pairs. The number of pairs corresponds to an integer bond order between two atomic centers, while the values of the correlations provide a fractional interpretation of these bonds.

Supplementary Note 3: Extended bonding analysis of the dissociation of LiH

In this section, we extend the bonding analysis of the LiH molecule. As pointed out in the main text, when the molecule is stretched beyond $R_{\mathrm{Li-H}} = 4$Å, the ground state no longer describes the realistic molecule, and instead the thermal state should be considered due to the closing gap between the singlet and triplet first excited states. Here, to elaborate more on this, we study in particular the behavior of the entanglement $E_{\mathrm{Li-H}}$ of individual energy eigenstates.

In Supplementary Figure 4, we present the energies of the ground and excited states from a density matrix renormalization group calculation with the aug-cc-pVDZ basis set, the delocalization index $\delta_{\mathrm{Li-H}}$ (taken from Ref. [2]), and the entanglement $E_{\mathrm{Li-H}}$ of the most entangled pair of MEAOs between the Li and H atoms in both the ground and first excited states. Around equilibrium, we still find a considerable amount of entanglement, $E \approx 0.77\log(4)$, in the ground state between a Li- and a H-MEAO. To understand how the level of ionicity is reflected in this entanglement

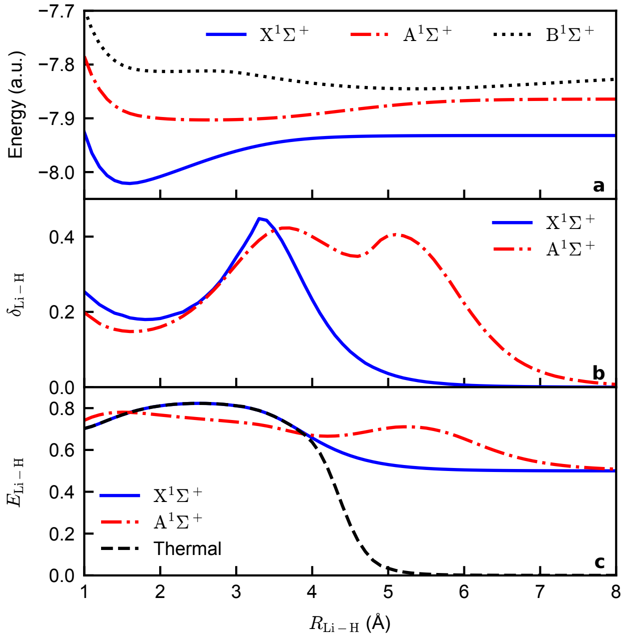

Suppl. Fig. 4. **LiH dissociation in the aug-cc-pVDZ basis.** **a.** Energies (in atomic unit, a.u.) of the lowest three states in the spin singlet sector ($X^1\Sigma^+$, $A^1\Sigma^+$, and $B^1\Sigma^+$). **b.** The electron delocalization index $\delta_{\mathrm{Li-H}}$ of the singlet ground and first exacted state. **c.** The highest entanglement value (normalized by log(4)) between two maximally entangled atomic orbitals, one localized on Li and one on H, in the singlet ground state, first excited state, and the thermal state at $\beta = 10^3\ \mathrm{Ha}^{-1}$ involving the singlet ground state and the triply degenerate triplet first excited states. Source data are provided as a Source Data file.

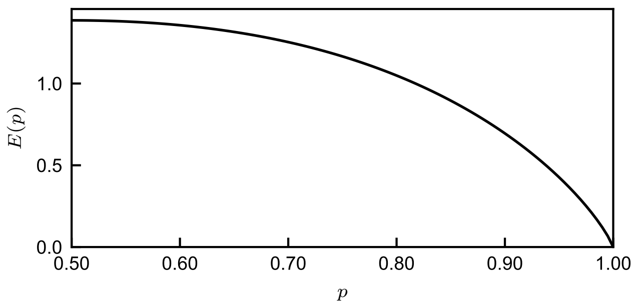

Suppl. Fig. 5. Orbital entanglement $E(p)$ as a function of parameter p in Eq. (13).

value, we consider the following family of parametrized states:

$$|\Psi(p)\rangle = p|0\rangle_L \otimes |\uparrow\downarrow\rangle_R + \sqrt{1-\frac{p^2}{3}}(|\uparrow\rangle_L \otimes |\downarrow\rangle_R - |\downarrow\rangle_L \otimes |\uparrow\rangle_R + |\uparrow\downarrow\rangle_L \otimes |0\rangle_R), \tag{13}$$

where p is the overlap between the state $|\Psi(p)\rangle$ and the fully ionic configuration. When $p = 1/2$, the state represents a perfect covalent bond, and when $p = 1$, the state is fully ionic. In Supplementary Figure 5, we present the entanglement between the left and right orbitals as a function of p. The entanglement changes slowly as the state $|\Psi(p)\rangle$ deviates from the perfect covalent state, explaining the relatively high level of entanglement in the ionic phase of LiH. According to this model, the entanglement value $E \approx 0.77 \log(4)$ corresponds to $p \approx 0.94$, indicating a highly ionic state.

In Supplementary Figure 4c, we observe a pronounced peak in $E_{\mathrm{Li-H}}$ in the ground state around the position of the avoided crossing at $R = 3$Å. After this, the entanglement decays with the dissociation and stabilizes at a value of 0.5. Note that the entanglement of the ground state does not decay to 0. This is because the electron spins on the Li and H atoms remain entangled due to the singlet symmetry of the ground state. However, this entanglement is

unphysical and unstable against thermal perturbation (as discussed in the main text) and should not be considered as an index for electron sharing. The entanglement in the first excited state displays a more intricate trend. After the first avoided crossing, the covalent first excited state becomes more ionic, which is correctly reflected by the decrease in $E_{\text{Li}-\text{H}}$. At $R_{\text{Li}-\text{H}} \approx 6\text{Å}$, another avoided crossing occurs between the first and second excited states, with the latter also exhibiting covalent character. Consequently, a similar effect is observed as in the ground state around $R_{\text{Li}-\text{H}} = 3\text{Å}$, namely a peak in the entanglement. This delicate change in the excited state was also observed in real-space partitioning [2] but has not been previously reported in Hilbert space partitioning.

Finally, we verify that the low-temperature thermal state provides a more realistic description of the molecule at all separation distances $R_{\text{Li}-\text{H}}$. In Supplementary Figure 4c, we overlay the entanglement $E_{\text{Li}-\text{H}}$ of the thermal state ($\beta = 10^3$) onto that of the ground state. When $R_{\text{Li}-\text{H}} < 4\text{Å}$, the entanglement of the thermal state and the ground state are identical. However, when $R_{\text{Li}-\text{H}} \geq 4\text{Å}$, the ground state remains unphysically entangled, whereas the thermal state correctly dissociates into unentangled fragments.

Supplementary Note 4: Other bonding indices

In this section, we provide the definitions of the bonding indices used in our article.

Electron delocalization index. Let A and B be two atomic regions in real space, defined by an atomic real-space partition. Given an electron density $\hat{\rho}(\mathbf{r})$, the electron population N_A in the atomic region A is calculated via the integral

$$N_A = \int_A \hat{\rho}(\mathbf{r})\,\mathrm{d}\mathbf{r}, \tag{14}$$

and likewise for N_B. Similarly, the pair population N_{AB} over the two atomic regions A and B is defined as the integral of the pair density $\hat{\rho}(\mathbf{r}_1, \mathbf{r}_2)$ [3]:

$$N_{AB} = \int_A \int_B \hat{\rho}(\mathbf{r}_1, \mathbf{r}_2)\,\mathrm{d}\mathbf{r}_1 \mathrm{d}\mathbf{r}_2. \tag{15}$$

The electron delocalization index δ_{AB} is then defined as the covariance of the populations between the atomic regions A and B, specifically as the discrepancy between the pair population and the product of the two atomic populations:

$$\delta_{AB} = 2(N_A N_B - N_{AB}) \equiv -2\mathrm{Cov}(N_A, N_B). \tag{16}$$

Aromatic fluctuation index. Let $\mathcal{A} = (A_1, A_2, \ldots, A_N)$ be a ring structure of N elements arranged in order along the ring. The aromatic fluctuation index $\mathrm{FLU}(\mathcal{A})$ is defined as [4]

$$\mathrm{FLU}(\mathcal{A}) = \frac{1}{N} \sum_{i=1}^{N} \left[\left(\frac{V_i}{V_{i-1}} \right)^{\alpha} \left(\frac{\delta_{A_i A_{i-1}} - \delta^{(\mathrm{ref})}_{A_i A_{i-1}}}{\delta^{(\mathrm{ref})}_{A_i A_{i-1}}} \right) \right], \tag{17}$$

where

$$V_i = \sum_{j \neq i} \delta_{A_i A_j}, \quad \text{and} \quad \alpha = \begin{cases} 1 & V_i > V_{i-1}, \\ -1 & V_i \leq V_{i-1}. \end{cases} \tag{18}$$

Here, $\delta^{(\mathrm{ref})}_{A_i A_{i-1}}$ is the reference value of the electron delocalization index for aromatic molecules. For example, $\delta^{(\mathrm{ref})}_{\mathrm{CC}} = 1.389$ for benzene [5] at the B3LYP/6-311G(d,p) level of theory. For aromatic rings, the value of FLU is close to 0.

Multicenter index. The first multicenter index for a ring structure $\mathcal{A} = (A_1, A_2, \ldots, A_N)$ was proposed by Giambiagi *et al.* [6]:

$$I_{\mathrm{ring}}(\mathcal{A}) = \sum_{i_1, i_2, \ldots, i_N} n_{i_1} n_{i_2} \cdots n_{i_N} S_{i_1 i_2}(A_1) S_{i_2 i_3}(A_2) \cdots S_{i_N i_1}(A_N), \tag{19}$$

where the indices $i_1, i_2, \ldots, i_N$ run through all natural orbitals, with n_k being the natural occupation numbers, and $S_{i_1 i_2}(A_1)$ the overlap of the two natural orbitals i_1 and i_2 over the atomic region A_1. If the system is described by a closed shell single Slater determinant, then the indices $i_1 i_2 \cdots i_N$ only run up to the number of occupied orbitals N_{occ}.

The value of I_{ring} depends on the ordering of the elements in the ring. To remove this dependency, the multicenter index MCI is defined as [7]

$$\text{MCI}(\mathcal{A}) = \frac{1}{2N} \sum_{\mathcal{P}(\mathcal{A})} I_{\text{ring}}(\mathcal{P}(\mathcal{A})), \tag{20}$$

where $\mathcal{P}(\mathcal{A})$ represents all possible permutations of the elements A_1, A_2, ..., A_N.

Nucleus-independent chemical shift. The nucleus-independent chemical shift (NICS) quantifies the intensity of the induced current on a ring structure under an external magnetic field [8, 9]. In this work, we employed the index NICS(1), where the magnetic probe is placed 1 Å above the molecular plane, avoiding contributions from σ-electrons.

Harmonic oscillator measure of aromaticity. The harmonic oscillator measure of aromaticity (HOMA) quantifies the level of aromaticity based on the molecular structure [10, 11]:

$$\text{HOMA} = 1 - \frac{\alpha}{N} \sum_{i=1}^{N} (R_{\text{opt}} - R_i)^2, \tag{21}$$

where N is the number of bonds, α is a bond-specific empirical constant (257.7 for C-C), R_i refers to the i-th bond length, and R_{opt} is the reference bond length of fully aromatic molecules.

[1] L. Ding, E. Matito, and C. Schilling, Maximally entangled atomic orbitals, https://doi.org/10.5281/zenodo.19553976 (2026).

[2] M. Rodríguez-Mayorga, E. Ramos-Cordoba, P. Salvador, M. Solà, and E. Matito, Bonding description of the harpoon mechanism, Mol. Phys. **114**, 1345 (2016).

[3] X. Fradera, M. A. Austen, and R. F. Bader, The Lewis model and beyond, J. Phys. Chem. A **103**, 304 (1999).

[4] E. Matito, M. Duran, and M. Sola, The aromatic fluctuation index (flu): A new aromaticity index based on electron delocalization, J. Chem. Phys. **122**, 014109 (2005).

[5] F. Feixas, E. Matito, J. Poater, and M. Solà, On the performance of some aromaticity indices: a critical assessment using a test set, J. Comp. Chem. **29**, 1543 (2008).

[6] M. Giambiagi, M. S. de Giambiagi, C. D. dos Santos Silva, and A. P. de Figueiredo, Multicenter bond indices as a measure of aromaticity, Phys. Chem. Chem. Phys. **2**, 3381 (2000).

[7] P. Bultinck, M. Rafat, R. Ponec, B. Van Gheluwe, R. Carbo-Dorca, and P. Popelier, Electron delocalization and aromaticity in linear polyacenes: atoms in molecules multicenter delocalization index, J. Phys. Chem. A **110**, 7642 (2006).

[8] P. v. R. Schleyer and H. Jiao, What is aromaticity, Pure Appl. Chem. **68**, 209 (1996).

[9] P. v. R. Schleyer, C. Maerker, A. Dransfeld, H. Jiao, and N. J. van Eikema Hommes, Nucleus-independent chemical shifts: a simple and efficient aromaticity probe, J. Am. Chem. Soc. **118**, 6317 (1996).

[10] J. Kruszewski and T. Krygowski, Definition of aromaticity basing on the harmonic oscillator model, Tetrahedron Lett. **13**, 3839 (1972).

[11] T. M. Krygowski, Crystallographic studies of inter-and intramolecular interactions reflected in aromatic character of. pi.-electron systems, J. Chem. Inf. Comput. Sci. **33**, 70 (1993).
