# Supplementary material for: Chemical bonding concepts emerge naturally from maximally entangled atomic orbitals
Source: Nat Commun. 2026 May 27;17:4732. doi: 10.1038/s41467-026-73527-w (PMC13216281; doi:10.1038/s41467-026-73527-w)
Supplement: Supplementary file 2 — Description of Additional Supplementary Files [file 41467_2026_73527_MOESM2_ESM.pdf]

Description of Additional Supplementary Files

Supplementary Data 1 | Unprocessed data and molecular structures (excluding available ones on the NIST database).
